# Intrauterine gestational diabetes mellitus, child anxiety, and internalizing symptoms: moderation by physical activity

**DOI:** 10.1210/jendso/bvag095

**Published:** 2026-04-20

**Authors:** Jasmin M Alves, Megan Herting, Ting Chow, Anny H Xiang, Kathleen A Page

**Affiliations:** Division of Endocrinology, Department of Medicine, Keck School of Medicine, University of Southern California, Los Angeles, CA 90033, USA; Diabetes and Obesity Research Institute, Keck School of Medicine, University of Southern California, Los Angeles, CA 90033, USA; Department of Population and Public Health Sciences, Keck School of Medicine, University of Southern California, Los Angeles, CA 90033, USA; Department of Research and Evaluation, Kaiser Permanente Southern California, Pasadena, CA 91101, USA; Department of Research and Evaluation, Kaiser Permanente Southern California, Pasadena, CA 91101, USA; Department of Health Systems Science, Kaiser Permanente Bernard J. Tyson School of Medicine, Pasadena, CA 91101, USA; Division of Endocrinology, Department of Medicine, Keck School of Medicine, University of Southern California, Los Angeles, CA 90033, USA; Diabetes and Obesity Research Institute, Keck School of Medicine, University of Southern California, Los Angeles, CA 90033, USA

**Keywords:** gestational diabetes mellitus, child mental health outcomes, child physical activity

## Abstract

**Context:**

Prenatal exposure to gestational diabetes mellitus (GDM) is linked to increased risk for offspring mental health disorders, yet few studies account for maternal mental health or potential protective factors.

**Objective:**

This work aimed to examine whether prenatal GDM exposure is associated with child anxiety and depressive symptoms, independent of maternal depression, and whether child physical activity (PA) modifies these associations.

**Methods:**

In this retrospective cohort study, data included 138 child-mother dyads (mean [SD] child age: 11.1 [2.5] years; 40% GDM-exposed; 56% female) from the BrainChild study. GDM status was extracted from electronic medical records. Children completed the State-Trait Anxiety Inventory for Children, Center for Epidemiological Studies Depression Scale for Children, Child Behavior Checklist, and a 3-day PA recall. Mothers completed the Center for Epidemiological Studies Depression Scale. Linear regression models, adjusted for child age, sex, puberty, and maternal depression, assessed associations between GDM exposure and mental health outcomes and tested moderation by moderate-to-vigorous physical activity (MVPA).

**Results:**

GDM-exposed children had higher parent-reported internalizing symptoms compared with unexposed children (β = 3.22; *P* < .01), independent of maternal depressive symptoms. MVPA significantly interacted with GDM exposure in such a way that the high MVPA group showed no association between GDM exposure and trait anxiety, while the low-MVPA group showed that GDM exposure was associated with higher trait anxiety (β = 4.11; *P* < .01).

**Conclusion:**

Prenatal GDM exposure was associated with greater internalizing symptoms during preadolescence, independent of maternal depression. Engagement in recommended PA levels mitigated the relationship between GDM-exposure and child anxiety, suggesting PA may protect against anxiety-related outcomes. Future prospective studies are needed to confirm these findings.

There has been an increase in mental health concerns among youth, with an urgent need to identify potential contributing factors [1, 2]. The developmental origins of health and disease hypothesis has highlighted that the brain is particularly sensitive to in utero insults that could potentially exacerbate one's risk of developing mental health disorders [3]. Notably, epidemiological studies have found that prenatal exposure to gestational diabetes mellitus (GDM) is associated with an increased risk for offspring developing anxiety and depressive disorders [4-7]. While there have been epidemiological studies identifying an increased risk of anxiety and depressive disorders among GDM-exposed offspring, limited studies have demonstrated associations with subclinical mental health symptoms such as anxiety and depressive symptoms, with the majority of studies completed in preschool-aged children [8-10]. Considering the onset of anxiety and depressive disorders occurs during adolescence, studying the preadolescent time period when anxiety and depressive symptoms begin to first manifest, is critical to identify potential mitigating factors [11].

A wealth of research has found that regular engagement in physical activity (PA) is associated with lower anxiety and depressive symptoms [12-16]. Randomized clinical trials in children have previously shown that aerobic exercise is effective in reducing anxiety and depressive symptoms [17-19]. Furthermore, longitudinal research has offered compelling evidence that PA in childhood serves as a protective factor against the development of adverse mental health outcomes [16, 20, 21]. Similarly, prior epidemiological studies found that PA modifies the relationship between perceived stress and depressive symptoms in children in such a way that children with high perceived stress who were more physically active reported fewer depressive symptoms [16, 22]. Additionally, our prior study showed that less time spent in vigorous PA during the height of the COVID-19 pandemic was related to heightened state anxiety among GDM-exposed children [23]. Therefore, we reasoned that PA could serve as a potential modifier of the relationship between GDM exposure and child anxiety and depressive symptoms. In contrast, maternal mental health concerns have been shown to negatively affect risk for anxiety and psychotic symptoms [24-26]. Yet, prior studies have often not accounted for the role of maternal mental health in the relationship between GDM exposure and child anxiety and depressive symptoms [5, 27-31]. Recent studies have suggested that maternal mental health increases the risk of GDM [32]; therefore, it is important to determine if the association between maternal GDM exposure and child anxiety and depressive symptoms is independent of maternal mental health.

Therefore the goals of this study were (1) to examine the relationship between GDM exposure and child anxiety and depressive symptoms, and the role of maternal mental health symptoms, using both self-reported and parent-reported symptoms of child anxiety and depression; and (2) to determine whether child PA modifies the relationship between prenatal exposure to GDM and child anxiety and depressive symptoms during the preadolescent time period when depressive and anxiety symptoms typically begin to first manifest [11]. We hypothesized that GDM exposure would be associated with increased anxiety and depressive symptoms independent of maternal mental health symptoms and that child PA would modify the relationship between prenatal exposure to GDM and anxiety and depressive symptoms.

## Materials and methods

### Study overview

BrainChild assesses the effects of prenatal exposure to GDM on child health outcomes; it is an ongoing longitudinal study where children participate in annual study visits. Children aged 7 to 11 years, were recruited for the BrainChild study starting in 2014, and baseline visits began in 2015. Children exposed to GDM were recruited first, followed by children unexposed to GDM, matched for child age, sex, and maternal prepregnancy body mass index (BMI). Mental health measures assessing anxiety and depression symptoms started in 2021. For this analysis, only each participant's first visit with mental health data were used for this report; therefore, a cross-sectional approach was used. Children were born at Kaiser Permanente Southern California (KPSC) and asked to participate in the study if they were free of any preexisting medical conditions and KPSC's electronic medical records (EMRs) have documentation of the mother's diagnosis of GDM or normal glucose tolerance during pregnancy. All children were of singleton uncomplicated births and were KPSC members at the time of birth. Study visits are completed at the University of Southern California. Parents provided consent for themselves and their child to participate in the study. Each child additionally provided assent. The institutional review boards at both KPSC (No. 10282) and the University of Southern California (USC) (No. HS-14-00034) approved this study.

### Exposure to gestational diabetes mellitus

Maternal GDM status was extracted from EMRs. GDM diagnosis was based on laboratory glucose values confirming a plasma glucose level of 11.10 mmol/L or greater from a 50-g glucose challenge tests or at least 2 plasma glucose values meeting or exceeding the following values on the 100-g or 75-g oral glucose tolerance test: fasting, 5.27 mmol/L; 1 hour, 9.99 mmol/L; 2 hours, 8.60 mmol/L; and 3 hours, 7.77 mmol/L (“Standards of Medical Care in Diabetes—2012,” 2012). Pregnant mothers at KPSC undergo routine glucose screening between 24 and 28 weeks of gestation, with earlier testing performed when clinically indicated: having a first-degree relative with diabetes, certain ethnic groups, previous history of delivering infant heavier than 4000 g, previous history of GDM, hypertension, high-density lipoprotein cholesterol level less than 35 mg/dL/a triglyceride level greater than 250 mg/dL, history of polycystic ovary syndrome, glycated hemoglobin A_1c_ greater than or equal to 5.7%, history of insulin resistance or cardiovascular disease [33].

Maternal prepregnancy BMI was calculated from maternal height (cm) and weight (kg) measurements closest to last menstrual period (LMP) from EMRs within 180 days before the last LMP or 90 days after the last LMP. Maternal height and weight measurements were collected during regular health visits and entered into EMRs by a health-care provider.

### Study measures

Child participants from the BrainChild study completed the State-Trait Anxiety Inventory for Children (STAIC) and the Center for Epidemiological Studies Depression Scale for Children (CES-DC) questionnaires. The STAIC has previously been validated in 1551 children and is used to measure self-reported state and trait anxiety [34]. State anxiety consists of 20 questions used to assess current feelings of anxiety while trait anxiety consists of 20 questions used to assess usual feelings of anxiety. The CES-DC includes 20 questions used to assess self-reported depressive symptoms for the past week and has been validated in children aged 6 to 23 years [35, 36]. Parents completed the Child Behavior Checklist (CBCL) for their child. The CBCL is validated both for clinical and research settings based on a sample of 4220 children [37, 38]. It consists of 113 questions that assess behavior in the past 6 months. For this study, we used the calculated scores for internalizing symptoms and externalizing symptoms. Internalizing symptoms are calculated based on the number of anxious/depressed symptoms, withdrawn/depressed symptoms, and somatic symptoms while externalizing symptoms are calculated based on the number of rule-breaking and aggressive symptoms. Examples of internalizing symptoms include being nervous, tense, or finding few things enjoyable. Examples of externalizing symptoms includes being disobedient at home or getting into fights. See the CBCL scoring manual for more details [37]. Mothers also completed the 20-item Center for Epidemiological Studies Depression Scale [39]. Higher scores were indicative of higher rates of depression.

The 3-Day Physical Activity Recall (3DPAR) is used to assess time spent in moderate and vigorous PA [40]. With the input of each child and child's parent, a trained staff member asks participants to recall their activities from 7 Am to 12 Am in 30-minute increments for the previous 3 days. The participant rates the intensity of each activity ranging from “light,” “moderate,” “hard” to “very hard.” Each activity and intensity level are converted to metabolic equivalences (METs) [41]. Activities with METs ≥3 (eg, bike riding, swimming) are classified as moderate-to-vigorous physical activity (MVPA). The final output is the daily average of minutes spent in MVPA. The 3DPAR is validated with accelerometers in pediatric populations [15, 40, 42, 43].

Tanner stage of puberty is determined from a validated questionnaire that is completed by the participant and their guardian to assess their Tanner stage of pubertal development [44, 45].

### Consent and approval

All data included in this study were obtained from participants whose parents gave their consent to participate in research after reading and signing an institutional review board (IRB)-approved consent form (USC IRB [No. HS-14-00034], KPSC IRB [No. 10282]). Children also provided assent to participate. This consent form covered all procedures carried out in the study as well as potential risks and discomforts.

### Statistical analysis

Participant characteristics were compared between unexposed and GDM-exposed children, using *t* tests for all continuous variables, and Fisher exact test for categorical variables. The primary outcomes of interest were self-reported anxiety (STAIC-trait) and depressive symptoms (CES-DC) and parent-reported symptoms of internalizing symptoms that are indicative of depressive and anxiety symptoms [34, 36, 46]. The secondary outcome of interest was externalizing symptoms, as we wanted to assess if GDM exposure was associated with externalizing symptoms in preadolescence similar to studies showing that GDM exposure was associated with externalizing symptoms in preschool-aged children [8, 28, 29]. Linear regression was used to assess associations between maternal depression and child mental health outcomes (self-reported and parent-reported anxiety and depressive symptoms) and externalizing symptoms. The first model was unadjusted. The second model was adjusted for child age, sex, Tanner stage of puberty, and maternal prepregnancy BMI. To assess associations between GDM exposure and child mental health outcomes (self-reported and parent-reported anxiety and depressive symptoms) and externalizing symptoms, 3 models were run using linear regression. The first model was unadjusted. The second model was adjusted for child age, sex, pubertal stage, and maternal prepregnancy BMI. The third model was further adjusted for maternal depressive symptoms. Interaction models were additionally tested to determine if the relationship between GDM exposure and child mental health outcomes differed by child PA levels. Stratified models were run for variables with statistically significant interactions. In stratified analyses, the same models were used. Child puberty was colinear with child age. Therefore, a yes-no categorical variable was created based on whether Tanner stage of puberty was prepubertal or postpubertal. Postpubertal was defined as a Tanner stage greater than 1. Child PA was made into a yes/no categorical variable, whether children engaged in the recommended amount of MVPA based on US Centers for Disease Control (CDC) recommendations. The CDC recommends that children engage in at least 60 minutes of MVPA a day [47]. Data from this analysis were collected from October 2021 to July 2025. Statistical significance was defined as *P* less than .05 for all analyses. For all analyses, SAS (SAS Institute) was used.

## Results

**Figure 1:**
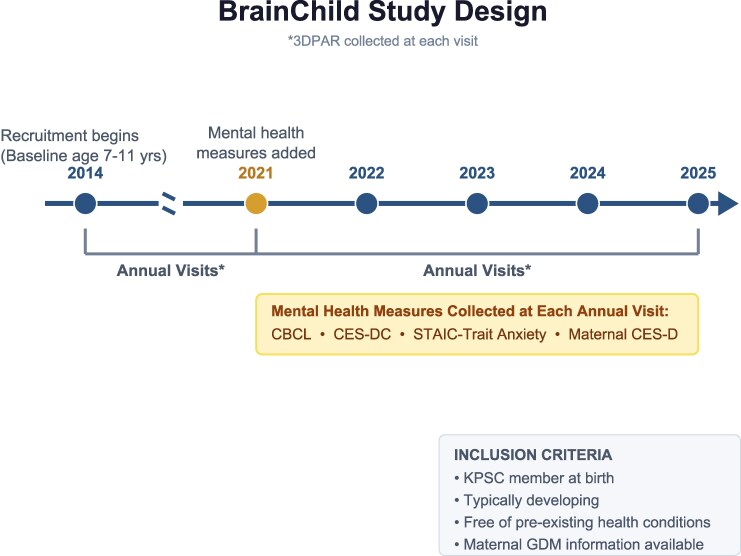
BrainChild Overview. Overview of BrainChild study design with mental health measures collected at each annual visit. 3DPAR, 3-Day Physical Activity Recall; CES-DC, Center for Epidemiological Studies Depression Scale for Children; CBCL, Child Behavior Checklist; STAIC, State-Trait Anxiety Inventory for Children; CES-D, Center for Epidemiological Studies Depression Scale.

Of the 251 participants with a baseline visit, 138 participants completed mental health measures (Fig. 1). Trait anxiety values were similar in the BrainChild Cohort compared to normative values [34] (STAIC mean [SD]: 37.32 [6.48] vs BrainChild mean (SD): 34.87 [6.98]). For internalizing symptoms, scores above 6.99 correspond to clinically significant (ie, above the threshold at which symptoms warrant clinical concern) for children aged 6 to 11 years [37]. Twenty-seven participants (20%) had internalizing scores in the clinically significant range (>10.99), while 31 participants (22%) had externalizing scores in the clinically significant range (>6.99). These percentages are higher than normative values from the CBCL, for which 2% of the sample used to create normative values had symptoms in the clinically relevant range [37]. Overall, 34% of BrainChild participants had depressive symptoms that may be indicative of clinical depression (CES-DC scores >15), which is similar to other studies of similarly aged children [48].

The results of the comparison between unexposed and GDM-exposed children are presented in Table 1. GDM-exposed children had significantly higher internalizing symptoms, trait anxiety, and age. Participants did not differ in other characteristics.

**Table 1:** Participant demographics

Variable	Unexposed children mean (SD) or N (%) (N = 83)	GDM-exposed children (SD) or N (%) (N = 55)	*P*
Maternal characteristics			
Prepregnancy BMI	30.16 (6.49)	31.72 (7.23)	.19
Maternal Depression (CES-D)	8.18 (7.45)	10.22 (7.32)	.12
50-g glucose challenge value (plasma glucose mg/dL)	112.5 (24.10)	154.0 (39.62)	<.01
GDM average wk of diagnosis	NA	23.84 (8.04)	NA
GDM early vs late diagnosis, wk			NA
<26	NA	21 (38%)	
≥26		34 (62%)	
GDM treatment			
Pharmacological treatment	NA	26 (47%)	NA
Lifestyle modification		29 (53%)	
Child characteristics			
Child age, y	10.70 (2.53)	11.80 (2.44)	.01
Child BMI	21.25 (6.34)	23.50 (6.10)	.04
CES-DC score depression scores	11.89 (7.82)	14.02 (9.19)	.15
CBCL-externalizing symptoms	4.71 (4.79)	5.36 (5.02)	.52
CBCL-internalizing symptoms	5.22 (4.90)	8.43 (6.17)	<.01
STAIC Trait Anxiety	33.87 (6.53)	36.42 (7.43)	.04
Tanner stage of development			.06
Tanner I	45 (54%)	16 (29%)	
Tanner II	9 (11%)	11 (20%)	
Tanner III	6 (7.0%)	6 (11%)	
Tanner IV	19 (23%)	16 (29%)	
Tanner V	3 (4%)	4 (7%)	
Unknown	1 (1%)	2 (4%)	
Child sex			.91
Male	37 (45%)	24 (44%)	
Female	46 (55%)	31 (56%)	
Met CDC MVPA guidelines			.50
Yes	53 (64%)	32 (58%)	
No	30 (36%)	23 (42%)	

Variables tested using *t* test for means and Fisher exact test for proportions. *P* values less than .05 denote statistical significance.

Abbreviations: BMI, body mass index; CBCL, Child Behavior Checklist; CDC, US Centers for Disease Control; CES-D, Center for Epidemiological Studies Depression Scale; CES-DC, Center for Epidemiological Studies Depression Scale for Children; MVPA, moderate to vigorous physical activity; STAIC, State-Trait Anxiety Inventory for Children.

Maternal depression was significantly associated with child depression (β = .31; 95% CI, 0.12-0.49; *P* < .01), externalizing symptoms (β = .19; 95% CI, 0.08-0.30; *P* < .01), internalizing symptoms (β = .30; 95% CI, 0.17-0.43; *P* < .01), and trait anxiety (β = .23; 95% CI, 0.07-0.38; *P* = .02) (Table 2). Adjusting for child age, sex, puberty, child BMI, and maternal prepregnancy BMI did not change these relationships.

**Table 2:** Relationship between maternal depression scores and child mental health outcomes

Outcome	β (95% CI)	*P*	Model
CES-DC depression scores	.31 (0.12-0.49)	**<.01**	Unadjusted
	.30 (0.12-0.48)	**<**.**01**	Fully adjusted
CBCL-externalizing symptoms	.19 (0.08-0.30)	**<**.**01**	Unadjusted
	.20 (0.09-0.32)	**<**.**01**	Fully adjusted
CBCL-internalizing symptoms	.30 (0.17-0.43)	**<**.**01**	Unadjusted
	.29 (0.16-0.42)	**<**.**01**	Fully adjusted
STAIC-Trait Anxiety	.23 (0.07-0.38)	**<**.**01**	Unadjusted
	.21 (0.05-0.36)	.**01**	Fully adjusted

Bolded values denote significant *P*-values at the threshold, *P* < .05. Models fully adjusted for child age, sex, Tanner stage of puberty, and maternal prepregnancy body mass index.

Abbreviations: CBCL, Child Behavior Checklist; CES-DC, Center for Epidemiological Studies Depression Scale for Children; STAIC, State-Trait Anxiety Inventory for Children.

**Figure 2:**
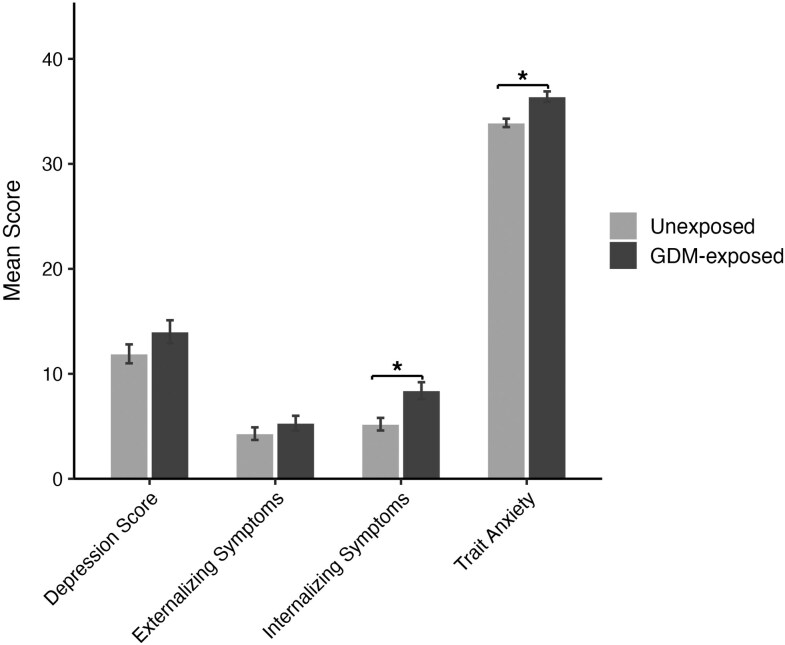
Mental health symptoms stratified by gestational diabetes mellitus (GDM) exposure. Bar plots reflect the means and standard error. Depression score comes from Center for Epidemiological Studies Depression Scale for Children (CES-DC), externalizing and internalizing symptoms come from the Child Behavior Checklist (CBCL), trait anxiety comes from the State-Trait Anxiety Inventory for Children.

GDM-exposed children had significantly higher internalizing symptoms than unexposed children (β = 3.22; 95% CI, 1.19-5.25; *P* < .01) (Table 3, Fig. 2). Further adjustment for child age, sex, puberty, child BMI, maternal prepregnancy BMI, and maternal depressive symptoms did not change these results. Before adjusting for covariates, GDM exposure was associated with higher trait anxiety (β = 2.55; 95% CI, 0.17-4.93; *P* = .04), but the relationship was not statistically significant in the adjusted models. Child depression and externalizing symptoms were not significantly different between unexposed and GDM-exposed children in either unadjusted or adjusted models. Further adjusting for maternal GDM treatment type did not affect results (Supplementary Table S1) [49].

**Table 3:** Association between gestational diabetes mellitus exposure and child mental health outcomes

Outcome	β (95% CI)	*P*	Model
CES-DC depression scores	2.13 (−0.73 to 4.99)	.15	Unadjusted
	1.33 (−1.61 to 4.26)	.38	Adjusted for child age, sex, Tanner stage of puberty, and maternal prepregnancy BMI
	.69 (−2.18 to 3.56)	.64	Further adjusted for maternal depression
CBCL-externalizing symptoms	1.04 (−0.7 to 2.77)	.24	Unadjusted
	.79 (−1.03 to 2.62)	.40	Adjusted for child age, sex, Tanner stage of puberty, and maternal prepregnancy BMI
	.36 (−1.4 to 2.12)	.69	Further adjusted for maternal depression
CBCL-internalizing symptoms	3.22 (1.19 to 5.25)	<.01	Unadjusted
	2.98 (0.87 to 5.08)	.01	Adjusted for child age, sex, Tanner stage of puberty, and maternal prepregnancy BMI
	2.39 (0.4 to 4.38)	.02	Further adjusted for maternal depression
STAIC-Trait Anxiety	2.55 (0.17 to 4.93)	.04	Unadjusted
	2.16 (−0.3 to 4.63)	.09	Adjusted for child age, sex, Tanner stage of puberty, and maternal prepregnancy BMI
	1.76 (−0.69 to 4.2)	.16	Further adjusted for maternal depression

Models fully adjusted for child age, sex, Tanner Stage of Puberty, maternal prepregnancy BMI, and maternal depression. Reference group is unexposed children.

Abbreviations: BMI, body mass index; CBCL, Child Behavior Checklist; CES-DC, Center for Epidemiological Studies Depression Scale for Children; GDM, gestational diabetes mellitus; STAIC, State-Trait Anxiety Inventory for Children.

**Figure 3:**
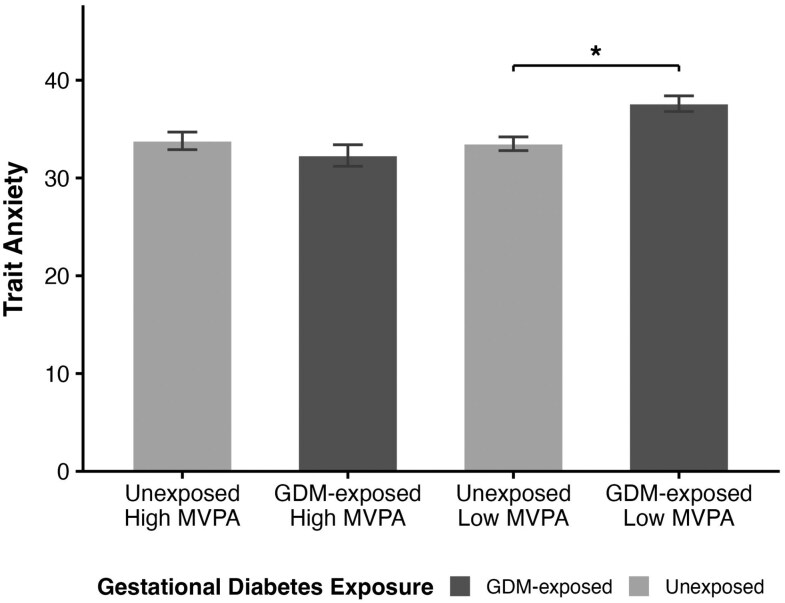
Mean (SE) of State-Trait Anxiety Inventory for Children (STAIC)-trait anxiety stratified by gestational diabetes mellitus (GDM) exposure and moderate to vigorous physical activity (MVPA) levels. Means for STAIC-Trait Anxiety unadjusted with SE bars.

There was a significant interaction between child MVPA and GDM exposure with child trait anxiety (*P* = .01). In analyses stratified by PA, GDM exposure was significantly associated with higher trait anxiety among the low-MVPA group, β = 4.11 (95% CI, 2.10-6.11; *P* < .01) (Fig. 3; Table 4). Further adjusting for covariates did not change this relationship. In the high-MVPA group, GDM exposure was unrelated to higher trait anxiety (*P* > .05).

**Table 4:** Association between moderate-to-vigorous physical activity and trait anxiety by gestational diabetes mellitus exposure

	Low MVPA		
Outcome	β (95% CI)	*P*	Model
STAIC-Trait Anxiety	4.11 (2.10 to 6.11)	**<.01**	Unadjusted
	3.78 (1.69 to 5.88)	**<.01**	Adjusted for child age, sex, Tanner stage of puberty, and maternal prepregnancy BMI
	3.74 (1.61 to 5.87)	**<.01**	Further adjusted for maternal depression

	High MVPA		
Outcome	β (95% CI)	*P*	Model
STAIC-Trait Anxiety	−1.56 (−4.30 to 1.19)	.27	Unadjusted
	−1.56 (−4.33 to 1.22)	.28	Adjusted for child age, sex, Tanner stage of puberty, and maternal prepregnancy BMI
	−2.05 (−4.80 to 0.69)	.15	Further adjusted for maternal depression

	Low MVPA	
Outcome	Beta (95% CI) *P*	Model
CES-DC	2.26 (−1.88 to 6.41) .27	Child age, puberty, and sex
Externalizing symptoms	1.38 (−4.14 to 6.90) .39	Child age, puberty, and sex
Internalizing symptoms	3.89 (−3.21 to 10.99) .14	Child age, puberty, and sex
State Anxiety	1.17 (−0.74 to 3.07) .21	Child age, puberty, and sex
Trait Anxiety	**4.39 (1.05 to 7.74) .01**	Child age, puberty, and sex

	High MVPA	
Outcome	β (95% CI) *P*	Model
CES-DC	−.11, (−4.32 to 4.11) .96	Child age, puberty, and sex
Externalizing symptoms	.68 (−3.25 to 4.61) .69	Child age, puberty, and sex
Internalizing symptoms	1.86 (−2.23 to 5.95) .31	Child age, puberty, and sex
State Anxiety	1.13 (−1.14 to 3.40) .30	Child age, puberty, and sex
Trait Anxiety	−1.99 (−5.37 to 1.32) .22	Child age, puberty, and sex

Bolded values denote significant *P*-values at the threshold, *P* < .05. Models fully adjusted for child age, sex, Tanner stage of puberty, maternal prepregnancy BMI, and maternal depression. Reference group is unexposed children.

Abbreviations: BMI, body mass index; CES-DC, Center for Epidemiological Studies Depression Scale for Children; GDM, Gestational Diabetes Mellitus; MVPA, moderate-to-vigorous physical activity; STAIC, State-Trait Anxiety Inventory for Children.

## Discussion

Prenatal exposure to GDM was associated with greater internalizing symptoms in preadolescent children, independent of maternal depression. Among GDM-exposed children, MVPA was associated with lower trait anxiety symptoms. Maternal depression was also related to child mental health outcomes, consistent with prior studies [24-26]. Collectively, these findings highlight prenatal exposure to GDM and child MVPA as important, interacting influences on anxiety and depressive symptoms during preadolescence, a sensitive developmental period.

Similar to epidemiological studies that have observed associations between anxiety and depressive disorders and GDM exposure, we found that prenatal exposure to GDM was associated with increased internalizing symptoms [6, 7]. This association was independent of maternal depression and maternal obesity. Numerous studies have neglected to account for maternal depression when assessing associations between GDM exposure and anxiety and depressive symptoms [27-30, 50]. Consistent with prior work [24-26], maternal depression was associated with child anxiety and depression in our sample. These findings emphasize the importance of accounting for maternal mental health when evaluating the effect of prenatal GDM exposure and child behaviors on mental health outcomes.

This is the first study to observe associations between prenatal exposure to GDM and internalizing symptoms during preadolescence after accounting for maternal depression and maternal obesity. Most prior studies have either focused on preschool-aged children or did not adjust for maternal depression [8, 10, 27-30, 50]. Interestingly, among preschoolers, only one study reported links between GDM exposure and increased internalizing symptoms [10], whereas most observed associations with externalizing symptoms [10, 28-30], likely reflecting developmental timing. Externalizing symptoms include behaviors such as hyperactivity and aggression, and typically emerge earlier in childhood [51], whereas internalizing symptoms, such as anxiety and depression, often begin to manifest in preadolescence [46]. Our findings may therefore capture early manifestations of internalizing symptoms not detectable at younger ages. Supporting this, large-scale epidemiologic studies have shown that the risk for psychiatric disorders following GDM exposure increases throughout childhood and peaks in adolescence [4-7]. Notably, Xiang et al (2024) [7] found the highest risk for an anxiety and depressive disorder in children exposed to GDM occurred during adolescence. Thus, our findings highlight the preadolescence as a critical window to identify and potentially intervene on emerging mental health vulnerabilities related to prenatal exposure to GDM.

Several intervention studies have shown that PA can reduce anxiety and depressive symptoms in adolescents [18, 52]. In our study, the relationship between GDM exposure and anxiety symptoms differed by PA levels, such that GDM exposure was unrelated to anxiety symptoms in the high MVPA but was associated with significantly higher anxiety symptoms in the low MVPA group. These findings suggest that PA during preadolescence could serve as a potential target for interventions to protect against the adverse consequences of prenatal exposure to GDM. Consistent with this, prior studies have found that PA confers particular benefits for vulnerable youth [22]. For example, in a study of 13 583 high school students, PA modified the relationship between depressive symptoms and a history of being bullied [53]. Additional longitudinal studies in youth have observed that PA protects against future symptoms of depression and anxiety [16, 54]. Together, this evidence suggests that engaging in PA may protect against adverse mental health outcomes among children at higher risk, including those prenatally exposed to GDM.

A notable strength of our study was the use of validated self-reported and parent-reported metrics for child mental health, complemented by objective confirmation of GDM exposure or nonexposure through EMRs. Limitations include a lack of objective PA measures (eg, accelerometers), and the correlational nature of the study. Furthermore, the lack of maternal mental health data during pregnancy and the lack of inclusion of additional health information that may have influenced maternal and child depressive symptoms such as vitamin D levels, represents a limitation of our study [55, 56]. Additional mechanistic studies are needed to test other factors that may have influenced maternal or child depressive symptoms. Moreover, future research is needed to experimentally determine if PA can reduce anxiety and depressive symptoms in GDM-exposed offspring and explore the mechanisms through which prenatal exposure to GDM affects offspring mental health outcomes. Additionally, longitudinal studies that extend into adolescence would provide valuable insight into the role of GDM exposure on mental health outcomes and how PA may act as a prevention in preadolescence. With the increasing prevalence of mental health issues in youth, it is imperative to identify modifiable behaviors that can improve mental health outcomes, particularly in vulnerable populations [1, 2].

### Conclusions

Our study demonstrates that prenatal exposure to GDM is associated with higher internalizing symptoms in preadolescence, independent of maternal prepregnancy BMI and maternal depression. Importantly, this is the first study to show that child PA may buffer the adverse effects of prenatal exposure to GDM on anxiety. Collectively, these findings highlight a nuanced relationship between GDM exposure, child anxiety, and depressive symptoms not typically assessed in epidemiological studies and underscore the need for longitudinal and intervention studies to confirm and extend these findings.

## Data Availability

Restrictions apply to the availability of some or all data generated or analyzed during this study to preserve patient confidentiality. The corresponding author will on request detail the restrictions and any conditions under which access to some data may be provided. See supplemental information at https://doi.org/10.17605/OSF.IO/A9TVS.

## Abbreviations

3DPAR: 3-Day Physical Activity Recall
BMI: body mass index
CBCL: Child Behavior Checklist
CDC: US Centers for Disease Control
CES-DC: Center for Epidemiological Studies Depression Scale for Children
EMR: electronic medical record
GDM: gestational diabetes mellitus
KPSC: Kaiser Permanente Southern California
LMP: last menstrual period
METS: metabolic equivalences
MVPA: moderate-to-vigorous physical activity
PA: physical activity
STAIC: State-Trait Anxiety Inventory for Children
